# Gene ontology analysis for RNA-seq: accounting for selection bias

**DOI:** 10.1186/gb-2010-11-2-r14

**Published:** 2010-02-04

**Authors:** Matthew D Young, Matthew J Wakefield, Gordon K Smyth, Alicia Oshlack

**Affiliations:** 1 Bioinformatics Division, The Walter and Eliza Hall Institute of Medical Research, 1G Royal Parade, Parkville 3052, Australia

## Abstract

We present GOseq, an application for performing Gene Ontology (GO) analysis on RNA-seq data. GO analysis is widely used to reduce complexity and highlight biological processes in genome-wide expression studies, but standard methods give biased results on RNA-seq data due to over-detection of differential expression for long and highly expressed transcripts. Application of GOseq to a prostate cancer data set shows that GOseq dramatically changes the results, highlighting categories more consistent with the known biology.

## Abstract 2

GOseq is a method for GO analysis of RNA-seq data that takes into account the length bias inherent in RNA-seq

## Background

Next generation sequencing of RNA (RNA-seq) gives unprecedented detail about the transcriptional landscape of an organism. Not only is it possible to accurately measure expression levels of transcripts in a sample [1], but this new technology promises to deliver a range of additional benefits, such as the investigation of alternative splicing [2], allele specific expression [3] and RNA editing [4]. However, in order to accurately make use of the data, it is vital that analysis techniques are developed to take into account the technical features of RNA-seq output. As many of the specific technical properties of RNA-seq data are not present in previous technologies such as microarrays, naive application of the same analysis methodologies, developed for these older technologies, may lead to bias in the results.

In RNA-seq experiments the expression level of a transcript is estimated from the number of reads that map to that transcript. In many applications, the expected read count for a transcript is proportional to the gene's expression level multiplied by its transcript length. Therefore, even when two transcripts are expressed at the same level, differences in length will yield differing numbers of total reads. One consequence of this is that longer transcripts give more statistical power for detecting differential expression between samples [5]. Similarly, more highly expressed transcripts have a greater number of reads and greater power to detect differential expression. Hence, long or highly expressed transcripts are more likely to be detected as differentially expressed compared with their short and/or lowly expressed counterparts. The fact that statistical power increases with the number of reads is an unavoidable property of count data, which cannot be removed by normalization or re-scaling. Consequently, it is unsurprising that this selection bias has been shown to exist in a range of different experiments performed using different analysis methods, experimental designs and sequencing platforms [5]. When performing systems biology analyses, failure to account for this effect will lead to biased results.

One simple, but extremely widely used, systems biology technique for highlighting biological processes is gene category over-representation analysis. In order to perform this analysis, genes are grouped into categories by some common biological property and then tested to find categories that are over represented amongst the differentially expressed genes. Gene Ontology (GO) categories are commonly used in this technique and there are many tools available for performing GO analysis - for example, EasyGO [6], GOminer [7], GOstat [8], GOToolBox [9], topGO [10], GSEA [11], DAVID [12] (see supplementary data 1 in Huang da *et al*. [12] for a more complete list). Although these tools have some differences in methodology [12], they all rely on similar underlying assumptions about the distribution of differentially expressed (DE) genes. Specifically, it is assumed that all genes are independent and equally likely to be selected as DE, under the null hypothesis. It is this assumption that makes the standard GO analysis methods unsuitable for RNA-seq data due to the bias in detecting differential expression for genes with a high number of reads. It follows then that when using a standard analysis, any category containing a preponderance of long genes will be more likely to show up as over-represented than a category with genes of average length. Similarly, categories with highly expressed genes are also more likely to be found as over-represented than categories of lowly expressed genes. Having identified this issue, it is possible to compensate for the effect of selection bias in the statistical test of a category's enrichment among differentially expressed genes.

This paper will be concerned with developing a statistical methodology that enables the application of GO analysis to RNA-seq data by properly incorporating the effect of selection bias. Using published RNA-seq data, we will show that accounting for this effect leads to significantly different results, which agree much better with previous microarray studies and the known biology than the results of an uncorrected analysis.

## Results

Because it is so widely used, we choose to focus our category testing on GO analysis. The techniques developed here apply more generally, however, to any analysis that looks for over-representation of some category of interest amongst differentially expressed genes. For example, alternative analyses might look for over-representation of KEGG (Kyoto Encyclopedia of Genes and Genomes) pathways, gene sets in the Molecular Signatures Database [11], or gene lists derived from earlier microarray experiments.

To illustrate the methodology, the GOseq technique was applied to an experiment examining the effects of androgen stimulation on a human prostate cancer cell line, LNCaP [13]. This published data set includes more than 17 million short cDNA reads obtained for both the treated and untreated cell line and sequenced on Illumina's 1G genome analyzer. These reads were re-mapped back to the reference genome and the number of reads per gene was recorded (see Additional file 1 for details). Examples of the methodology throughout the paper are made using this data set and we show that the GOseq method makes a substantial difference to the results of the GO analysis, which are more consistent with prior knowledge of the system.

### GO analysis

Standard methods for testing over-representation of a GO category assume that, under the null hypothesis, each gene has equal probability of being detected as DE. Under this assumption, the number of genes associated with a category that overlap with the set of DE genes follows a hypergeometric distribution. Hence the GO test can be conducted using Fisher's exact test, which uses the hypergeometric distribution, or Pearson's chi-square test, which is a computationally convenient approximation [12]. Because of the existence of selection bias, genes with more reads are more likely to be detected as DE, violating the assumption behind the hypergeometric distribution. Therefore, these standard test distributions should not be used for GO analysis with RNA-seq data. Instead, we need a new test that corrects for selection bias.

### Selection bias

**Figure 1:**
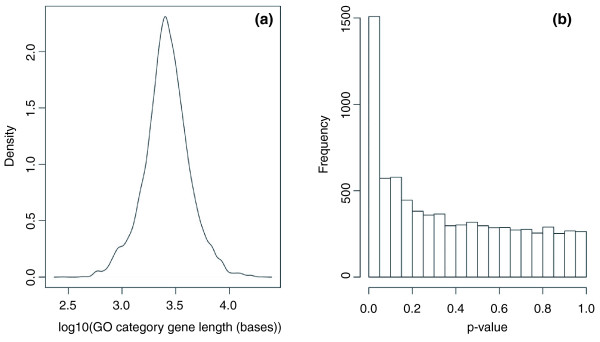
**Length distribution of genes in Gene Ontology categories**. **(a) **The distribution of average gene lengths in GO categories on a log10 scale. The GO category gene length is given by the median length of the genes within the category. **(b) ***P*-values for the two-sided Mann-Whitney U test comparing the median length of genes in a GO category with the overall distribution of genes for 7,873 GO categories. The excess of low *P*-values shows that there are many GO categories that contain a set of significantly long or short genes.

Transcript length bias will affect GO analysis if the sets of genes associated with GO categories contain a non-random set of genes, with either a preponderance of short or long genes. To test individual categories for a length distribution bias, we assessed the length of genes within 7,873 unique GO categories associated with human genes in the GO consortium database [14]. The length distribution of the genes in these categories varied widely, with some categories containing an over-representation of long genes and some with relatively short genes (Figure 1a). A Mann-Whitney U test (also known as a Wilcoxon rank-sum test) was performed on each category to test whether the median length of genes in that GO category differed from the global median length across all categories. Figure 1b shows a histogram of the Mann-Whitney *P*-values. The clear excess of low *P*-values indicates the existence of GO categories with many long or short genes. Therefore, we expect that a standard analysis of GO category enrichment among DE genes, which ignores transcript length, to be significantly affected by the transcript length bias inherent to RNA-seq.

### The GOseq method

In order to correct for selection bias in category testing, we propose the following three-step methodology. First, the genes that are significantly DE between conditions are identified. The GOseq method works with any procedure for identifying DE genes. Second, the likelihood of DE as a function of transcript length is quantified. This is obtained by fitting a monotonic function to DE versus transcript length data. Finally, the DE versus length function is incorporated into the statistical test of each category's significance. This final step takes into account the lengths of the genes that make up each category.

**Figure 2:**
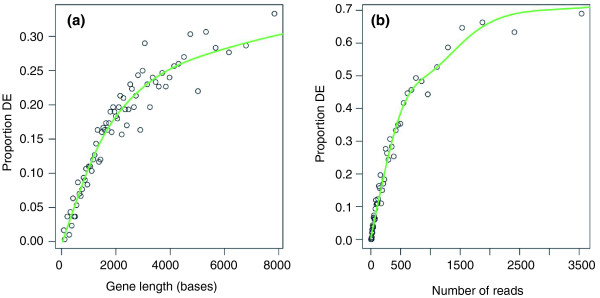
**Differential expression as a function of gene length and read count**. **(a) **The proportion of DE genes is plotted as a function of the transcript length. Each point represents the percentage of genes called DE in a bin of 300 genes plotted against the median gene length for the bin. The green line is the probability weighting function obtained by fitting a monotonic cubic spline with 6 knots to the binary data. A clear trend towards more detected differential expression at longer gene lengths can be seen. **(b) **The same, except instead of transcript length, the total number of reads for each gene was used. Again, a trend towards more DE for genes with more reads can be seen. Note the greater range of probabilities compared to (a).

The first step in the GOseq procedure is to determine which genes are differentially expressed. For the prostate cancer data set, a *P*-value for differential expression between the treated and untreated cells was obtained for each gene using a Poisson exact test, equivalent to Fisher's exact test [15-17]. These *P*-values were then corrected for multiple testing [18] and the false discovery rate was set at 10^-4^. Figure 2a shows a plot of the proportion of DE genes as a function of length. A strong trend towards a higher rate of differential expression for genes with longer transcripts is evident. Figure 2b shows a similar trend towards more differential expression for genes with a higher total number of reads. As expected, the trend is stronger for total read count compared to transcript length. Other statistical methods for determining differential expression between samples show a similar trend of increasing proportion of DE genes with gene length, even when working with data normalized by dividing by transcript length, such as RPKM transformed data [19] (Figure S1b,c in Additional file 1). Non-statistical methods for determining DE, such as using a fold-change cutoff, can show a decreasing trend in the proportion of DE as a function of gene length (Figure S1a in Additional file 1). Any trend observed in the data is modeled by the second step of the GOseq procedure.

In the second step, a probability weighting function (PWF) is estimated from the data. The PWF quantifies how the probability of a gene selected as DE changes as a function of its transcript length. To estimate this trend function, each gene is assigned a binary value (zero or one), according to whether or not the gene is found to be DE. A monotonic spline with 6 knots is then fitted to this binary data series using the transcript length of each gene as predictor (see Materials and methods). Monotonicity is imposed as the power to detect DE using statistical tests increases monotonically with an increasing number of reads. Figure 2 also shows the resulting probability weighting function for the prostate cancer data set. The PWF then forms the null hypothesis for our enrichment test.

Unlike GO analysis for microarray data, the null probability distribution does not conform to a standard distribution, precluding an analytical solution for determining the probability of a category being over-represented among DE genes. However, the *P*-value for each category can be computed using a resampling strategy. For the final step of the GOseq method, resampling was performed by randomly selecting a set of genes, the same size as the set of DE genes, and counting the number of genes associated with the GO category of interest. This random selection weights the chance of choosing a gene by its length or read count, from the previously fitted probability weighting function. The resampling is repeated many times and the resulting distribution of GO category membership is taken to approximate the shape of the true probability distribution. This sampling distribution allows calculation of a *P*-value for each GO category being over-represented in the set of DE genes while taking selection bias into account.

### The Wallenius approximation

Although accurate, random sampling is very computationally intensive, particularly when a fine granularity in *P*-value is needed to distinguish between large numbers of categories. In order to make the calculation in the final step of the GOseq procedure computationally manageable, we implemented an alternative approximation technique, based on an extension of the hypergeometric distribution known as the Wallenius non-central hypergeometric distribution [20]. This distribution extends the hypergeometric distribution to the case where the probability of success and failure differ. The GOseq implementation of the approximation assumes that all genes within a category have the same probability of being chosen, but this probability is different from the probability of choosing genes outside this category. The mean of the probability weightings for each gene within/outside the category is defined as the common probability of choosing a gene within/outside that category. While the Wallenius approximation is obviously a simplification, it is significantly closer to the true distribution than the standard hypergeometric distribution.

Although the accuracy lost by using the Wallenius approximation is not negligible, the gain in computational efficiency is dramatic. Furthermore, the ability to differentiate the most highly over-represented categories from one another (Additional file 1) makes the Wallenius approximation an attractive alternative, particularly when the range of the probability weighting function is moderate.

### Comparisons of GOseq with the standard GO analysis

**Figure 3:**
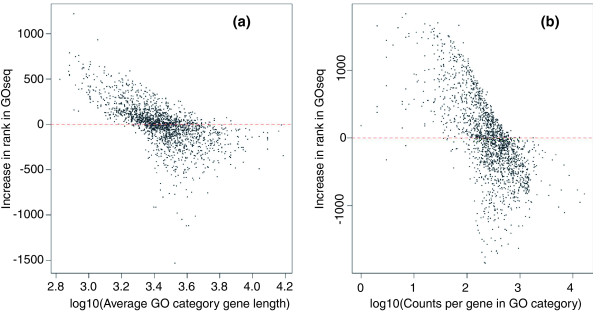
**Change in Gene Ontology category rank between the standard and GOseq methodologies**. **(a) **Change in rank of GO categories going from the hypergeometric method to GOseq correcting for length bias plotted against the log of the average gene length of the category. **(b) **Change in rank of GO categories going from the hypergeometric method to GOseq correcting for total read count plotted against the log of the average number of counts of each gene in the category. A trend for the standard method to underestimate significance for GO categories containing short (or highly expressed) genes and overestimate significance for GO categories containing long (or underexpressed) genes can be clearly seen.

To compare the results from the GOseq procedure to the standard GO analysis used on microarray data, we applied both methods to the prostate cancer data set. For each method a list of GO categories ordered by significance was generated. Figure 3 compares the ranks of the GO categories between the GOseq method and hypergeometric method as a function of gene length (Figure 3a) or total read count (Figure 3b) within categories. As expected, categories with shorter than average length move up in rank (become more significant) when length bias has been taken into account with the GOseq method. Similarly, categories with longer than average genes are ranked lower by GOseq than the standard method. Furthermore, when accounting for gene length bias, of the 25 categories most significantly over-represented using GOseq and the standard method, 8 are discrepant between the methods (Tables 1 and 2). This highlights the fact that accounting for biases in detecting DE makes a significant difference to the biology identified from the results.

**Table 1:** Gene Ontology categories ranked in the top 25 in the standard method but not in length bias adjusted GOseq

GOID	Term	Ontology	Rank standard	Rank GOseq	Average gene length in category
GO:0005622	Intracellular	CC	9	38	2,710
GO:0005524	ATP binding	MF	12	113	3,133
GO:0008270	Zinc ion binding	MF	13	145	2,817
GO:0016020	Membrane	CC	16	702	2,571
GO:0046872	Metal ion binding	MF	17	255	2,734
GO:0006355	Regulation of transcription, DNA-dependent	BP	19	266	2,752
GO:0000139	Golgi membrane	CC	22	28	2,855
GO:0016740	Transferase activity	MF	25	116	2,721

The median gene length of all categories are significantly longer than average using a Mann-Whitney test. BP, biological process; CC, cellular component; MF, molecular function.

**Table 2:** Gene Ontology categories ranked in the top 25 in length bias adjusted GOseq but not in the standard method

GOID	Term	Ontology	Rank standard	Rank GOseq	Average gene length in category
GO:0006414	Translational elongation	BP	92	8	708*
GO:0000786	Nucleosome	CC	64	14	1,345*
GO:0006334	nucleosome assembly	BP	70	17	1,362*
GO:0043687	Post-translational protein modification	BP	50	20	1,830
GO:0019787	Small conjugating protein ligase activity	MF	65	25	1,928
GO:0016192	Vesicle-mediated transport	BP	28	22	2,765
GO:0006412	Translation	BP	104	23	1,472
GO:0051246	Regulation of protein metabolic process	BP	66	24	1,609

Three of these categories (marked with asterisks) have genes significantly shorter than average length (Mann-Whitney *P*-value < 0.05). BP, biological process; CC, cellular component; MF, molecular function.

**Figure 4:**
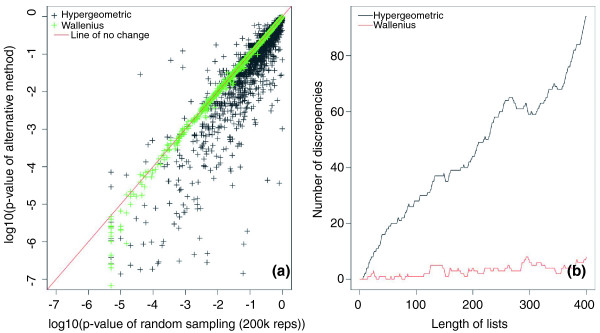
**Comparison of GOseq and the standard hypergeometric methods**. **(a) **The *P*-values generated with GOseq using the Wallenius approximation and the standard hypergeometric method are plotted against the *P*-values calculated with GOseq using random sampling (200k repeats). The Wallenius method (green crosses) shows good agreement with the high resolution (200,000 repeats) random sampling. A large discrepancy in *P*-values is seen between GOseq and the hypergeometric method. **(a) **The number of discrepancies between lists is shown for a given list size. The black line compares GOseq using high resolution sampling with the hypergeometric method. The red line compares GOseq using high resolution sampling with the Wallenius approximation. Again, GOseq using the Wallenius method shows little difference from GOseq using the random sampling method (with 200k repeats) while the hypergeometric method shows a large number (approximately 20%) of discrepancies.

The standard GOseq analysis was also compared to both the random sampling strategy and the more computationally efficient Wallenius approximation. *P*-values for over-representation of DE genes for each GO category were generated using random sampling with a high number of repetitions (200,000). These *P*-values were then compared to *P*-values calculated using the standard hypergeometric test and GOseq utilizing the Wallenius approximation. Comparison of the categories' *P*-values demonstrate a large discrepancy between the GOseq method and the hypergeometric method, as well as very little difference between GOseq using sampling or Wallenius (Figure 4a; Figure S3 in Additional file 1). Furthermore, we compared the top ranked lists of enriched GO categories between two methods by plotting the number of discrepancies between the methods for a given list size (Figure 4b). This plot shows that for the prostate cancer data set, approximately 20% of GO categories that appear using the standard analysis are not present when GOseq is used and vice versa. The high number of discrepancies for short lists shows that failure to account for length bias impairs analysis, even if we are only interested in a small number of categories. Reassuringly, the Wallenius approximation closely approximates GOseq using high repetition sampling with very few changes in *P*-values or rankings of categories. Similar results are seen for other RNA-seq datasets (data not shown).

### Measuring GOseq's accuracy by comparison with microarray data

**Figure 5:**
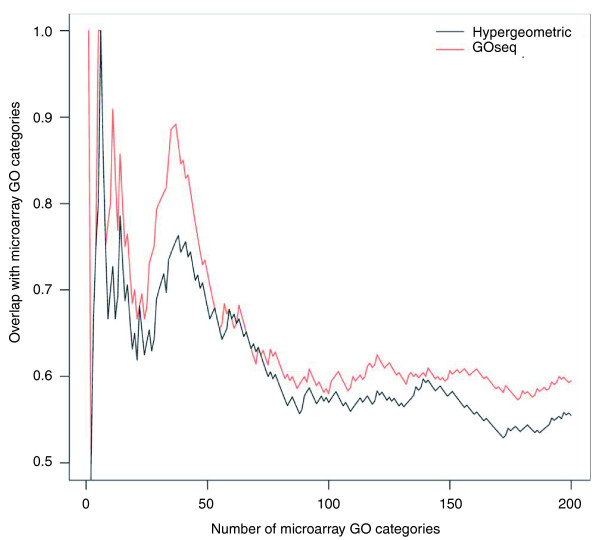
**A comparison of Gene Ontology analysis using RNA-seq and microarrays on the same samples**. The fraction of GO categories identified by RNA-seq data that overlap with the microarray GO analysis are shown as a function of the number of categories selected. RNA-seq data have been analyzed using GOseq and hypergeometric methods. The GOseq categories have a consistently higher overlap with the microarray GO categories than the standard method.

The GOseq method clearly makes a substantial difference to the categories selected when performing a GO analysis. In order to demonstrate that accounting for length bias produces more reliable results, we compared the results of GOseq and the standard test to the GO analysis from a microarray experiment that does not show any gene length bias [5]. For the comparison of RNA-seq and microarrays, a data set was used that compares the exact same liver and kidney samples on the two platforms [21] (see Materials and methods). Figure 5 plots the fraction of microarray GO categories recovered from the RNA-seq data using the hypergeometric and GOseq methods, as a function of the number of GO categories considered. It can be seen that GOseq gives categories more consistent with the microarray platform (*P *= 0.067), indicating that accounting for length bias gives a GO analysis with better performance.

### Transcript length bias versus read count bias

As transcript length bias is a technical effect, it is always necessary to correct for it when performing category testing on RNA-seq data. However, the bias in power to detect DE in longer genes arises from an increase in the total number of reads for each gene, where the number of reads is given by transcript length multiplied by expression level. Therefore, there may be circumstances where it is desirable to correct for the effect of expression level on power to detect DE, in addition to the contribution from transcript length, that is, total read count bias (for further discussion see Additional file 1). The GOseq method is capable of handling both types of bias. We have mainly focused on transcript length bias as it will always need to be accounted for and because the decision to account for expression level or not ultimately depends on the questions the user wishes to answer.

**Figure 6:**
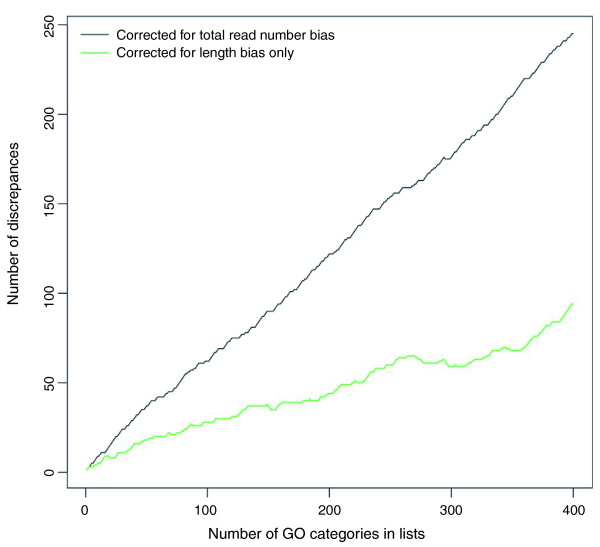
**Change in most significant categories when correcting for total read count bias or length bias versus the standard method**. A plot of the number of discrepancies in the most significant GO categories generated using different methods. This plot compares the length bias correcting version of GOseq to the standard hypergeometric method (green line) and the total read count bias correcting version of GO-seq to the standard hypergeometric method (black line).

To assess the impact of total read count bias, GOseq was used to analyze the prostate cancer data set accounting for read count bias. As expected, correcting for read count bias results in even greater differences compared to the standard hypergeometric method than just correcting for length bias alone. When read count bias is corrected for in the prostate cancer data set, more than 50% of significant GO categories are different from the list of significant GO categories obtained using the standard hypergeometric method (Figure 6). This is true even for the very top GO categories - there is only one GO category that appears in the top ten in both the standard and read count adjusted lists (Tables 3 and 4).

**Table 3:** Top ten Gene Ontology categories using the standard method

GOID	Term	Ontology	Rank standard	Rank GOseq	Median read count of genes in category
GO:0005515	Protein binding	MF	1	11	206
GO:0005737	Cytoplasm	CC	2	111	238
GO:0005634	Nucleus	CC	3	4,303	228
GO:0000166	Nucleotide binding	MF	4	2,546	252
GO:0005829	Cytosol	CC	5	1,192	349
GO:0005783	Endoplasmic reticulum	CC	6	6	197
GO:0003677	DNA binding	MF	7	2,109	189
GO:0005794	Golgi apparatus	CC	8	26	213
GO:0005622	Intracellular	CC	9	3,592	187
GO:0016874	Ligase activity	MF	10	88	353

BP, biological process; CC, cellular component; MF, molecular function.

**Table 4:** Top ten Gene Ontology categories using GOseq adjusted for total read count bias

GOID	Term	Ontology	Rank standard	Rank GOseq	Median read count of genes in category
GO:0016020	Membrane	CC	16	1	91
GO:0005886	Plasma membrane	CC	96	2	50
GO:0005887	Integral to plasma membrane	CC	313	3	36
GO:0005576	Extracellular region	CC	4,310	4	18
GO:0015020	Glucuronosyl transferase activity	MF	388	5	10
GO:0005783	Endoplasmic reticulum	CC	6	6	197
GO:0016021	Integral to membrane	CC	179	7	68
GO:0006470	Protein amino acid dephosphorylation	BP	31	8	224
GO:0045944	Positive regulation of transcription from RNA polymerase II promoter	BP	39	9	133
GO:0007049	Cell cycle	BP	14	10	202

BP, biological process; CC, cellular component; MF, molecular function.

## Discussion

### Biological relevance of selected categories

To determine the effect of GOseq on the ability to draw biologically meaningful conclusions, the top ten GO categories using the standard hypergeometric method and the read count adjusted GOseq method were compared in the prostate cancer data set (Tables 3 and 4). We found that categories identified by GOseq are more consistent with previous studies looking at the relationship of androgens with prostate cancer. The role of androgens in prostate cancer is well supported, with androgen required in rodent prostate cancer induction models, and castration prior to puberty being protective against prostate cancer. Androgen is thought to be responsible for promotion of prostate cancer progression through enhancing the androgen regulated processes of growth and cellular activity. In normal prostate, androgen supports the secretary epithelial, which turns over at a rate of 1 to 2% of cells per day, and most prostate cancers are derived from these cells [22]. Based on this biological knowledge the prior expectation is that there will be an increase in cellular activity, proliferation and secretion in LNCaP cells in response to androgen. Previous microarray experiments have shown that LNCaP cells retain androgen responsiveness and that most genes upregulated are involved in the production of seminal fluid [23].

In the standard analysis, the top ten GO categories indicate a change in intracellular genes, including nuclear and DNA binding genes (Table 3). The top ten categories using GOseq with total read count adjustment indicate significant changes at membranes and extracellular space, transcriptional upregulation, and in cell cycle genes (Table 4). The categories identified as most significant by GOseq therefore better match the known biology of androgen response in prostate cancer. The genes that are significant only with the length bias adjustment (Table 2) include four categories consistent with increased translation and protein production, vesical mediated transport consistent with secretion, and two categories related to nucleosomes consistent with increased replication. The category of small conjugating protein ligase activity is supported by the previously reported up-regulation of ubiquitin ligases UBE2C and HSPC150 [23].

### Possibility of true biological trends in gene length

A key assumption of GOseq is that longer genes are not of biologically greater interest than shorter genes, *per se*. This assumption is supported by microarray data, where no systematic trend between gene length and differential expression has been observed [5]. The authors find it hard to imagine that any genuine biological process could induce a trend in differential expression versus gene length comparable in magnitude to the technical trend that is removed by GOseq. Nevertheless, users should be aware that any biological trend in DE versus gene length will be adjusted for by GOseq.

### Using the Wallenius approximation

The reduction in computation afforded by the Wallenius approximation is predicated on the assumption that the variance in the probability weighting within categories is low. Hence, the approximation will perform better for a probability weighting function with a low range of probabilities. When the range in probabilities as a function of gene length or read count is large, random sampling performs better than the Wallenius approximation, even at a relatively small number of replicates. The probability weighting function for read count bias has a larger range in probabilities compared to the PWF for length bias in the prostate cancer data set (Figure 2) and so random sampling may be more appropriate when accounting for total read count bias.

### GOseq and other technologies

GOseq with total read count adjustment is relevant for other tag-based next generation expression profiling technologies, such as SAGE or CAGE, which have no transcript length bias [24]. Even without length bias, statistical power to detect differential expression still depends on the expression level of each transcript and correcting for this bias will generally be desirable in this context.

GO analysis of microarray expression data has so far ignored the possibility of selection bias, but such bias clearly does exist. It is well known that fold changes are more precisely estimated for microarray probes at higher intensity levels, so selection bias is likely to exist as a function of intensity. Furthermore, most microarray platforms have multiple probes for some genes. Genes with more probes will have a great chance of being selected as DE, assuming the analysis is conducted probe-wise. The methodology developed here for RNA-seq could easily be adapted to GO analysis of microarray data, and would likely yield benefits in terms of biological relevance.

### GOseq software

In order to implement the GOseq method, we developed a set of freely available R functions, which includes functions for calculating the significance of over-representation of each GO category amongst DE genes. These functions give the user the option of selecting which type of bias they wish to compensate for (transcript length bias or total read count bias). The option of using random sampling or the Wallenius approximation is also available. The ability of the user to supply their own categories for unbiased testing is also included. The software is freely available and can be downloaded from our website [25].

## Conclusions

Here we have developed a statistical framework for GO analysis for use with RNA-seq data. It is mathematically indisputable that all commonly used criteria for judging DE interact with gene length and read count. This provides a well understood causal model of why length bias exists and why it needs to be accounted for. The GOseq method is able to account for such biases when performing GO analysis. We find that the new method makes a substantial difference to the categories identified as the most significant. We show that the GOseq method is able to recover well established microarray results more readily than existing methods of GO analysis of RNA-seq data. Furthermore, using an androgen treated prostate cancer data set we find that the most significant categories identified using GOseq match the known biology better than existing methods.

## Materials and methods

All the statistical analyses were performed in R [26]. The methods are described in detail in Additional file 1 and outlined briefly here.

### The prostate cancer data set

The LNCap cell line was treated with androgen. Mock treated and treated cell lines were sequenced using the Illumina GA 1 [13]. Raw 35-bp RNA-seq reads were provided and mapped to the human genome using Bowtie. Each mapped read was associated with an ENSEMBL gene. A Poisson exact test [15,16] was used to determine differential expression between treated and mock-treated LNCap cells.

### The liver versus kidney data set

Genome-wide expression was measured in liver and kidney using RNA-seq on the Illumina GA I and hybridization of the same samples to Affymetrix HG-U133 Plus 2.0 arrays. The sample preparation and data analysis was designed to maximize the similarity between the microarray and RNA-seq experiments (see Marioni *et al*. [21]). Differential expression between kidney and liver was determined using an empirical Bayes modified t-statistic on the microarray platform and *P*-values for DE were downloaded from their website. For the RNA-seq experiment, the data were normalized using TMM normalization [27] and a negative binomial exact test was used to determine DE [16]. To test the GOseq method, we used the genes called DE from the microarray experiment to calculate the significance of over-representation of each GO category using the standard GO analysis methods. We also calculated *P*-values for each GO category being over-represented among genes that were DE in the RNA-seq data, using both the GOseq and hypergeometric methods. GOseq's ability to outperform the hypergeometric method, as measured by its ability to reproduce the results of the microarray GO analysis, was quantified by calculating a *P*-value for the difference in the two methods being due to chance. To do this, a NULL was chosen under which both methods were equally likely to correctly recover each microarray GO category, with this likelihood given by a binomial distribution.

### The probability weighting function

To calculate the PWF, a cubic spline with a montonicity constraint is fitted to the binary data series where a value of 1 refers to a DE gene and 0 refers to a non-DE gene. This fit can be calculated against either the length of the gene or the read counts of a gene. We used the R function pcls in the mgcv package to generate the fit.

### Calculating significance of categories

Random samples of genes are created by selecting a subset of genes from the experiment, with each gene weighted by the probability derived from the PWF. Each random sample contains the same number of genes as the set of DE genes. For each sample the number of genes with a given GO category is calculated. Many samples are generated in order to produce a null distribution from which the *P*-value for the significance of a category can be estimated.

To implement the Wallenius approximation, we used the BiasedUrn package in R. The 'odds' parameter is defined as the relative probability of genes within a category to the genes outside the category. The 'odds' ratio is calculated by taking the mean of the values from the PWF for each gene in the set and dividing by the mean of the values from the PWF for genes outside the set.

## Abbreviations

DE: differentially expressed; GO: Gene Ontology; PWF: probability weighting function.

## Authors' contributions

AO, GKS, and MJW conceived of the idea. AO, MDY, and GKS conceived and performed the analysis. MDY wrote the software. MJW interpreted biological results. MDY, MJW, GKS, and AO wrote the paper. All authors read and approved the final manuscript.

## Supplementary Material

Additional file 1 A Word document containing supplementary methods, discussion and results. Particular attention is given to technical details of the GOseq method. Click here for file

## References

[B1] Fu X Fu N Guo S Yan Z Xu Y Hu H Menzel C Chen W Li Y Zeng R Khaitovich P Estimating accuracy of RNA-Seq and microarrays with proteomics. BMC Genomics 2009 10 161 19371429 10.1186/1471-2164-10-161 PMC2676304

[B2] Wang ET Sandberg R Luo S Khrebtukova I Zhang L Mayr C Kingsmore SF Schroth GP Burge CB Alternative isoform regulation in human tissue transcriptomes. Nature 2008 456 470 476 18978772 10.1038/nature07509 PMC2593745

[B3] Wang X Sun Q McGrath SD Mardis ER Soloway PD Clark AG Transcriptome-wide identification of novel imprinted genes in neonatal mouse brain. PloS One 2008 3 e3839 19052635 10.1371/journal.pone.0003839 PMC2585789

[B4] Wahlstedt H Daniel C Enstero M Ohman M Large-scale mRNA sequencing determines global regulation of RNA editing during brain development. Genome Res 2009 19 978 986 19420382 10.1101/gr.089409.108 PMC2694479

[B5] Oshlack A Wakefield MJ Transcript length bias in RNA-seq data confounds systems biology. Biol Direct 2009 4 14 19371405 10.1186/1745-6150-4-14 PMC2678084

[B6] Zhou X Su Z EasyGO: Gene Ontology-based annotation and functional enrichment analysis tool for agronomical species. BMC Genomics 2007 8 246 17645808 10.1186/1471-2164-8-246 PMC1940007

[B7] Zeeberg BR Feng W Wang G Wang MD Fojo AT Sunshine M Narasimhan S Kane DW Reinhold WC Lababidi S Bussey KJ Riss J Barrett JC Weinstein JN GoMiner: a resource for biological interpretation of genomic and proteomic data. Genome biology 2003 4 R28 12702209 10.1186/gb-2003-4-4-r28 PMC154579

[B8] Beissbarth T Speed TP GOstat: find statistically overrepresented Gene Ontologies within a group of genes. Bioinformatics 2004 20 1464 1465 14962934 10.1093/bioinformatics/bth088

[B9] Martin D Brun C Remy E Mouren P Thieffry D Jacq B GOToolBox: functional analysis of gene datasets based on Gene Ontology. Genome Biol 2004 5 R101 15575967 10.1186/gb-2004-5-12-r101 PMC545796

[B10] Alexa A Rahnenfuhrer J Lengauer T Improved scoring of functional groups from gene expression data by decorrelating GO graph structure. Bioinformatics 2006 22 1600 1607 16606683 10.1093/bioinformatics/btl140

[B11] Subramanian A Tamayo P Mootha VK Mukherjee S Ebert BL Gillette MA Paulovich A Pomeroy SL Golub TR Lander ES Mesirov JP Gene set enrichment analysis: a knowledge-based approach for interpreting genome-wide expression profiles. Proc Natl Acad Sci USA 2005 102 15545 15550 16199517 10.1073/pnas.0506580102 PMC1239896

[B12] Huang da W Sherman BT Lempicki RA Systematic and integrative analysis of large gene lists using DAVID bioinformatics resources. Nat Protoc 2009 4 44 57 19131956 10.1038/nprot.2008.211

[B13] Li H Lovci MT Kwon YS Rosenfeld MG Fu XD Yeo GW Determination of tag density required for digital transcriptome analysis: application to an androgen-sensitive prostate cancer model. Proc Natl Acad Sci USA 2008 105 20179 20184 19088194 10.1073/pnas.0807121105 PMC2603435

[B14] Ashburner M Ball CA Blake JA Botstein D Butler H Cherry JM Davis AP Dolinski K Dwight SS Eppig JT Harris MA Hill DP Issel-Tarver L Kasarskis A Lewis S Matese JC Richardson JE Ringwald M Rubin GM Sherlock G Gene ontology: tool for the unification of biology. The Gene Ontology Consortium. Nat Genet 2000 25 25 29 10802651 10.1038/75556 PMC3037419

[B15] Robinson MD Smyth GK Moderated statistical tests for assessing differences in tag abundance. Bioinformatics 2007 23 2881 2887 17881408 10.1093/bioinformatics/btm453

[B16] Robinson MD Smyth GK Small-sample estimation of negative binomial dispersion, with applications to SAGE data. Biostatistics 2008 9 321 332 17728317 10.1093/biostatistics/kxm030

[B17] Robinson MD McCarthy DJ Smyth GK edgeR: a Bioconductor package for differential expression analysis of digital gene expression data. Bioinformatics 2009 26 139 140 19910308 10.1093/bioinformatics/btp616 PMC2796818

[B18] Benjamini Y Hochberg Y Controlling the false discovery rate: a pratical and powerful approach to multiple testing. J R Stat Soc Series B (Methodological) 1995 57 289 300

[B19] Mortazavi A Williams BA McCue K Schaeffer L Wold B Mapping and quantifying mammalian transcriptomes by RNA-Seq. Nat Methods 2008 5 621 628 18516045 10.1038/nmeth.1226 PMC13303166

[B20] Wallenius KT Biased sampling: the non-central hypegeometric probability distribution. 1963 PhD: Stanford University

[B21] Marioni JC Mason CE Mane SM Stephens M Gilad Y RNA-seq: an assessment of technical reproducibility and comparison with gene expression arrays. Genome Res 2008 18 1509 1517 18550803 10.1101/gr.079558.108 PMC2527709

[B22] Feldman BJ Feldman D The development of androgen-independent prostate cancer. Nat Rev Cancer 2001 1 34 45 11900250 10.1038/35094009

[B23] DePrimo SE Diehn M Nelson JB Reiter RE Matese J Fero M Tibshirani R Brown PO Brooks JD Transcriptional programs activated by exposure of human prostate cancer cells to androgen. Genome Biol 2002 3 RESEARCH0032 12184806 10.1186/gb-2002-3-7-research0032 PMC126237

[B24] t Hoen PA Ariyurek Y Thygesen HH Vreugdenhil E Vossen RH de Menezes RX Boer JM van Ommen GJ den Dunnen JT Deep sequencing-based expression analysis shows major advances in robustness, resolution and inter-lab portability over five microarray platforms. Nucleic Acids Res 2008 36 e141 18927111 10.1093/nar/gkn705 PMC2588528

[B25] GOseq software http://bioinf.wehi.edu.au/software/goseq/

[B26] The R Project for Statistical Computing http://www.r-project.org/

[B27] Robinson MD Oshlack A A scaling normalization method for differential expression analysis of RNA-seq data. Genome Biology 2010 in press 10.1186/gb-2010-11-3-r25 PMC2864565 20196867

[B28] Langmead B Trapnell C Pop M Salzberg SL Ultrafast and memory-efficient alignment of short DNA sequences to the human genome. Genome Biol 2009 10 R25 19261174 10.1186/gb-2009-10-3-r25 PMC2690996

[B29] Ensembl BioMart http://www.biomart.org

[B30] Barnard GA Contribution to the discussion of Professor Bartlet's paper. J Roy Stat Soc Series B (Methodological) 1963 25 294

[B31] Efraimidis P Spirakis PG Weighted random sampling with a reservoir. Information Processing Lett 2005 97 181 185

[B32] Smyth GK Gentleman R, Care V, Dudoit S, Irizarry R, Huber W Limma: linear models for microarray data. Bioinformatics and Computational Biology Solutions using R and Bioconductor 2005 New York: Springer 397 420

