# Supplementary material for: Gene ontology analysis for RNA-seq: accounting for selection bias
Source: Genome Biol. 2010 Feb 4;11(2):R14. doi: 10.1186/gb-2010-11-2-r14 (PMC2872874; doi:10.1186/gb-2010-11-2-r14)
Supplement: Additional file 1 — A Word document containing supplementary methods, discussion and results. Particular attention is given to technical details of the GOseq method. [file gb-2010-11-2-r14-S1.doc]

# Additional file - Supplementary material

# Methods

**The Prostate Cancer Data Set**

For each sample we were provided with the raw 35 bp RNA-seq reads from the authors. For the untreated prostate cancer cells (LNCaP cell line) there were 4 lanes totaling ~10 million, 35 bp reads. For the treated cells there were 3 lanes totaling ~7 million, 35 bp reads. All replicates were technical replicates. Reads were mapped to NCBI version 36.3 of the human genome using bowtie [28]. Any read with which mapped to multiple locations was discarded. Using the ENSEMBL 54 annotation from biomart [29], each mapped read was associated with an ENSEMBL gene. This was done by associating any read that overlapped with any part of the gene (not just the exons) with that gene. Reads that did not correspond to genes were discarded. To find genes for which the number of mapped reads differed significantly between samples (that is, the differentially expressed genes), we used the exactTestNB() function with r=1e6 in the edgeR package to calculate a p-value based on a Poisson exact test [16, 17]. These p-values were then corrected for multiple hypothesis testing with a Benjamini-Hochberg adjustment [18]. Any gene meeting the FDR cutoff of 10^-4^ was marked as being DE.

**Obtaining GO categories**

For each gene we obtained a list of all associated GO categories, as well as the lengths (in bp) of all its transcripts using the ENSEMBL annotation obtained from biomart [29]. We took the length of a gene to be the median length of all its transcripts. The length of each transcript was given by the number of bp in its cDNA transcript.

## Comparing Microarray and RNA-seq data from Marioni et al.

As a test of the GOseq method, we made use of data from an existing experiment [21] designed to compare the RNA-seq and microarray platforms. For this data set, genome wide expression was measured in liver and kidney using RNA-seq on the Illumina GA I & hybridization of the same samples to Affymetrix HG-U133 Plus 2.0 arrays. The sample preparation and data analysis was designed to maximize the similarity between the microarray and RNA-seq experiments (see Marioni et al. 2008). Differential expression between kidney and liver was determined using an empirical Bayes modified t-statistic on the microarray platform and p-values for DE were downloaded from their website. For the RNA-seq experiment, the data was normalized using TMM normalization [27] and a negative binomial exact test was used to determine DE [16]. In order to use these data to test the GOseq method we used the genes called DE from the microarray experiment to calculate the significance of over representation of each GO category using standard GO analysis methods. Next we calculated p-values for each GO category being over representation among genes which were DE in the RNA-seq data, using both the GOseq and hypergeometric methods. By taking the microarray GO analysis as our gold standard, we compared the ability of GOseq and existing methods to reproduce these results. We plot the fraction of recovered microarray GO categories as a function of the number of GO categories considered in Figure 5 of the main paper.

**Calculating the Probability Weighting Function (PWF)**

To obtain a probability weighting function describing how percentage DE changes as a function of gene length, we generate a fit to the binary series given by each gene’s differential expression (1=DE, 0=not DE) as a function of each gene’s length (or read count). As the functional form of this dependence is unknown, we chose to use a cubic spline. Given that power for detecting DE increases with length (or read count), we impose a monotonicity constraint on our fit. The fit uses the penalized constrained least squares technique, implemented in the mgcv R library. A fit is obtained using a monotonic cubic spline with 6 knots. We tested other functional forms for this fit, such as a generalized linear model, but they were found to perform more poorly at the extremes of the distribution. The resulting procedure for creating the fit is exactly the same for total read counts, where we fit the binary DE series to total read counts for each gene.

**Calculating enrichment using the PWF**

Having obtained a probability weighting function, a resampling scheme is used to generate p-values taking into account selection bias. We generate a random set of DE genes of the same size as our detected set of DE genes. To ensure our random set has the same bias towards genes with high read counts, we weight the probability of each gene being chosen by its corresponding value on the PWF found earlier. This procedure is repeated many times and the number of times each GO category is found in our random sample is tallied. A p-value for over-representation of a category is then given by x+1/S+1, where x is the number of samples with as many or more genes associated with the GO category of interest and S is the total number of random sets [30]. Using this technique, it should be possible to achieve arbitrary accuracy given enough samples. In order to perform this random sampling as quickly as possible, a C sub-routine was written. This routine uses the weighted random sampling without replacement algorithm described in Efraimidis, 2005 [31]. We calculated 200,000 random samples, which we took to be accurate enough to represent the true probability of a GO category being over represented amongst DE genes (our truth data set).

Given infinite computation time, the random sampling method, outlined above, will accurately account for the effect of transcript length bias in RNA-seq data. However, in any real world application computational power will obviously be limited. At best, S random samples allow us to calculate the probability of over representation to a precision of 1/(S+1) (that is all probabilities have an uncertainty of at least 1/(S+1)). For example, a probability of 1/(S+1) determined by random sampling means the p-value is <1/(S+1). The end result is that, even on a modern cluster, the time taken to calculate p-values to the accuracy necessary to adequately differentiate GO categories from one another is often prohibitive.

For the prostate cancer data set used in this paper, computing the p-values for 7629 GO categories using random sampling with 200,000 repetitions took approximately 40 minutes. The calculation was performed on a MacBook Pro running OS X 10.5.7, with a 2.53 GHz Intel Core 2 Duo processor and 4 GB of RAM.

# Results

The discrepancies between the GOseq method and the hypergeometric methods are not artifacts of sample size or homogeneity of categories but in fact account for the gene length or read count biases. In order to show this we plotted the change in rank between the GO testing methods as a function of the number of genes in a category and the standard deviation of gene length in each GO category (Figure S1). There is no significant effect due to category size or variance evident in either plot.


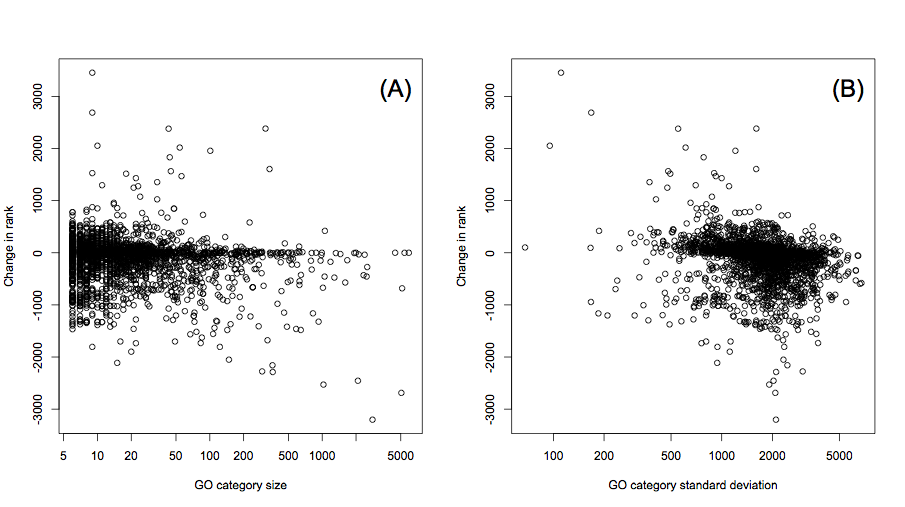


**Figure S1:** The change in rank of each category between the GOseq and hypergeometric methods are shown as a function of (A) number of genes in a category and (B) the standard deviation of gene lengths within a category.

## Alternative methods for determining DE

Although we use a Poisson exact test to determine DE, GOseq will work with any DE methodology. To illustrate this, we used three additional methods to determine differentially expressed genes in the prostate cancer data set. The first method calls all genes with a log2 fold change in number of reads greater than 3 as DE. The second method only counts reads lying within the exon of a gene. The number of reads is then divided by the total number of reads for each sample and the length of exonic sequence within each gene and multiplied by 10^9^ giving RPKM transformed data (reads per kilobase of exon model per million mapped reads) [19] for each gene. As a test for DE is not specified by Mortazavi A, et al. 2008 we log2 transformation the RPKM data and used Limma [32] to fit a linear model followed by an empirical Bayes adjustment of the variances and defined a FDR cutoff of 10% to call genes as DE. The third method we considered used a negative binomial distribution to model the counts for each gene and determine if the different in counts between samples was statistically significant [16]. This is similar to the Poisson method we used for our main analysis, but with an additional parameter to account for over dispersion.

Each of these methods for determining differential expression makes different assumptions about the data and as such produce very different lists of DE genes. Obviously, using the appropriate DE method is of vital importance in obtaining biologically meaningful results from an RNA-seq analysis. However, our motivation for considering different tests here is to illustrate that these different approaches do not remove the selection bias for detecting DE. As such, we will not discuss the validity of the different methods.

For each of the three methods, a plot of average gene length vs fraction DE was produced and a PWF was calculated, as can be seen in Figure S2. As expected, a gene length trend is still present for all DE methods and the GOseq procedure is able to account for this effect by fitting a PWF in each case. The PWFs all have different shapes and scales because each method returned sets of DE genes with different size and composition.

It is clear from Figure S2 that different methods for determining DE can result in significantly different trends of proportion DE vs. gene length. Most strikingly, using a fold change cutoff to determine DE, results in a decreasing, rather than increasing, trend as gene length increases. This is because chance variation is relatively large for genes with fewer reads, so large fold changes are more likely by chance, especially when the transcript has zero or very few counts in one of the conditions.

More generally, the exact shape of the PWF cannot be predicted in advance. This underscores the necessity for estimating the PWF from the whole genome. It is essential that the PWF reflect the technical trend present in the actual biological data under consideration and the DE methodology being used.


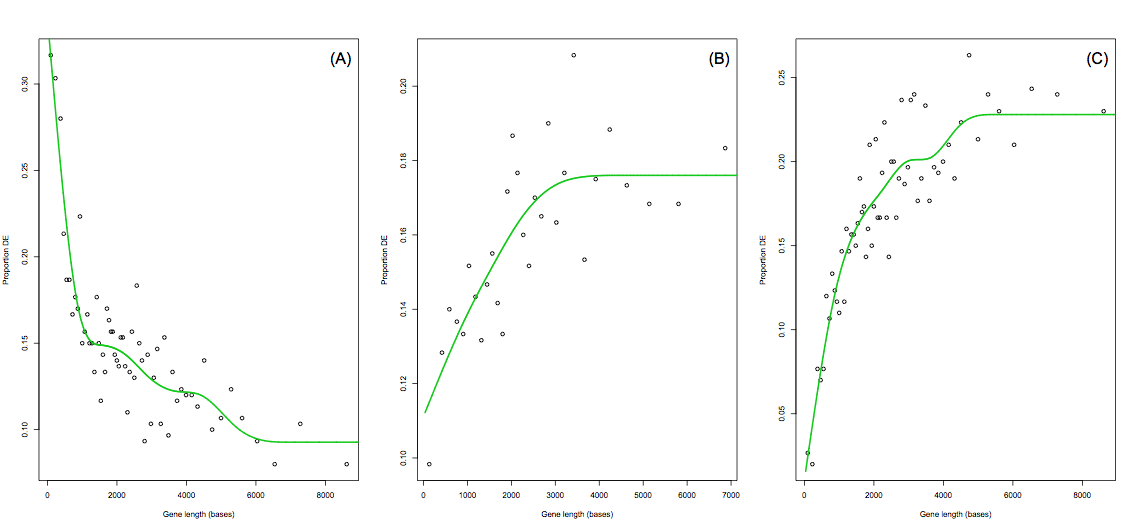


**Figure S2 - Probability Weighting Functions for alternative differential expression tests.** Proportion DE plotted against gene length for the Li et al. (2008) data for three different DE methods: (A) log2 fold change cutoff greater than 3, (B) limma test on RPKM normalized data, (C) negative binomial exact test. A strong length bias can be seen regardless of which statistical test is used.

**Using the Wallenius distribution to approximate GO category enrichment**

We have shown in Figure 4 of the paper that GOseq using the Wallenius approximation corresponds well to the random sampling method. Figure S3 below again shows the effectiveness of the approximation by plotting both the p-values for GOseq using the random sampling method and the Wallenius approximation against the standard hypergeometric method. It can be seen that the two GOseq methods coincide very well and are both quite different from the standard method.


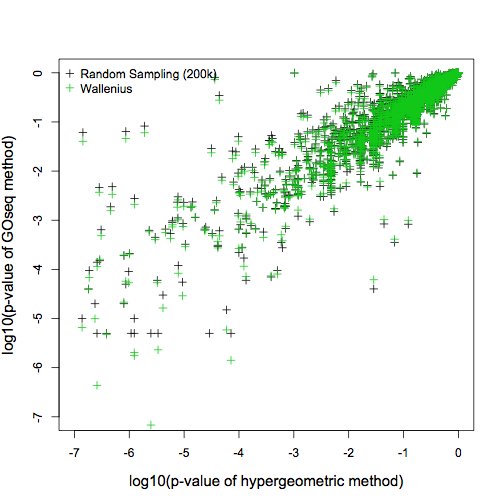


**Figure S3 – Accuracy of the Wallenius approximation as compared to random sampling with many repeats.** The p-values for over representation of DE genes in each GO category are obtained using, GOseq with random sampling (green/black points) and the Wallenius aprroximation (green/black points) and are plotted against the hypergeometric p-values. It can be seen that the Wallenius approximation reproduces random sampling very accurately in most cases.

The Wallenius distribution is used to approximate the probability of a category being over represented amongst DE genes. This approximation makes the simplifying assumption that genes with the GO category of interest have the same probability of being chosen and this probability is different from the probability of choosing the other genes. In order to use the Wallenius distribution, the probabilities for the two groups needs to be calculated. The Wallenius distribution is then completely determined by one parameter, the ratio of the two groups’ probabilities. We evaluated several methods for calculating the probability weights by determining their difference from the results of random sampling. Table S1 summarizes the different methods attempted as well as their relative accuracies as measured by the squared difference p-values on a log scale. We found the most accurate way to calculate the weighting is to first fetch the associated probability weighting (using the probability weighting function) of each gene, as determined by its transcript length, and then take the mean across the category of those values.

## Table S1 - Comparison of methods for calculating probabilities for use with the Wallenius approximation

| **Measure Name** | **Description** | **Agreement with true p-values based on squared difference in log p-values** |
| --- | --- | --- |
| Mean then fetch | For a given GO category, get all the associated genes. Take the mean of their gene lengths. Then get the value of the probability weighting function for this value. | .155 |
| Fetch then mean | For a given GO category, get all the associated genes. For each of these genes, get the corresponding value on the probability weighting function. Take the mean of these values. | .022 |
| Median then fetch | For a given GO category, get all the associated genes. Take the median of their gene lengths. Then get the value of the probability weighting function for this value. | .103 |
| Fetch then median | For a given GO category, get all the associated genes. For each of these genes, get the corresponding value on the probability weighting function. Take the median of these values. | .103 |

A further advantage of the Wallenius approximation over random sampling is that it is an analytical distribution and therefore p-values calculated form it are continuous and unaffected by granularity, giving the resolution to distinguish categories with very similar p-values. By contrast, even for our simulation with 200,000 repeats, the top 28 GO categories all have the minimum p-value.

Random sampling takes roughly the same amount of time to test any number of categories, with any number of DE genes. On the other hand, the time taken to perform the Wallenius approximation varies linearly with the number of categories being tested. So while ~8000 GO categories takes roughly the same time to test with 2500 random samples or the Wallenius approximation, testing 10 categories is two orders of magnitude faster with the Wallenius approximation, while the time for random sampling remains roughly the same. This makes the Wallenius approximation particularly attractive when the number of categories to be tested is small. In our implementation there is no appreciable variability in calculation time with the number of DE genes for either method.

Using the Wallenius approximation in adjusting for total read count bias is less accurate than the Wallenius approximation for length bias (Figure S4). However it is still a dramatic improvement on the standard method.


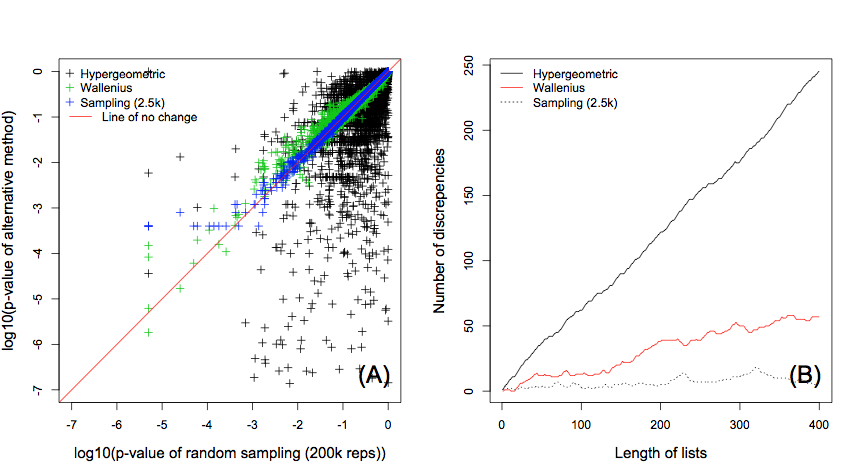


**Figure S4 - The accuracy of the Wallenius approximation and random sampling with a low number of counts when correcting for read count bias using GOseq.** (A) The p-values generated with the Wallenius, hypergeometric and random sampling (2.5k repetitions) methods, plotted against the p-values calculated using random sampling (200k repeats). The Wallenius method (green crosses) do not show such good agreement with the “truth” (taken to be random sampling with 200k repeats). In this case, random sampling with 2.5k repeats (blue crosses) is more accurate. (B) The number of discrepancies between lists of the most significant GO categories, comparing the “truth” data set to Hypergeometric, Wallenius and low number of samples. Again, the Wallenius approximation performs worse than low-repetition sampling, although it is still a marked improvement over the hypergeometric method.

# Discussion

## Other sources of RNA-seq bias

The majority of other biases in RNA-seq are dependent on the precise processing methodology. Some effects such as the censoring of products smaller than the library size selection, fragmentation bias, 3’ bias introduced by polyA selection and 5’ bias introduced by random initiation of reverse transcription have the potential to reduce the effective size of the transcript by making a portion of the transcript underrepresented in the data. We have chosen not to explicitly include these features as they are difficult to predict a priori, but are implicitly modeled by the read count bias approach.

## Further discussion of transcript length bias vs. read count bias

There are a number of reasons why correcting for total read count bias may be desirable. Adjusting for total read count has the effect of adjusting for each gene’s expression level in addition to its transcript length. Differential expression analysis of RNA-seq data based on p-values is likely to detect many genes at higher read counts with small fold-changes and genes with lower read counts will need larger fold-changes to achieve the same significance level. Correcting for total read count bias, which gives more weight to genes with lower counts, will tend to give more weight to genes with higher fold changes. Higher fold changes often translate to greater biological significance. For example, giving more weight to lower expressed, high fold-change transcripts increases the likelihood of detecting functional categories like transcription factors, which don’t have to be expressed at high levels, and gives less weight to house-keeping genes, which are often too ubiquitous to be of interest.

Although read count bias may improve detection of changes in lowly expressed genes, it incorporates expression level, which is a distinctly biological property. Unlike transcript length, which is an inherent property of the gene and independent of the biology, read count varies between conditions. Removing read count bias might serve to obscure, rather than clarify, the biological differences being investigated. If high expression is itself of interest then adjusting for transcript length alone is preferred.

Ultimately, the decision to test for either read count bias or length bias should be determined by what is relevant to the user’s research. If the aim is to find categories over represented in their experiment, regardless of the gene length, then GOseq should adjust for length bias only. Alternatively, the user may aim to find categories that are over represented, regardless of gene length or expression level, in which case the analysis should adjust for total read count bias. In both situations the researcher should consider the limitations and weighting given to different classes of genes when interpreting the output of GOseq.
